# Organ Regeneration Through Stem Cells and Tissue Engineering

**DOI:** 10.7759/cureus.34336

**Published:** 2023-01-29

**Authors:** Laiba Ajmal, Sidra Ajmal, Maleeha Ajmal, Gul Nawaz

**Affiliations:** 1 Chemistry and Biochemistry, University of Oklahoma, Norman, USA; 2 Biochemistry, University of Oklahoma, Norman, USA; 3 Internal Medicine, Marshfield Clinic Health System, Marshfield, USA

**Keywords:** extracellular matrices, engineering techniques, stem cell therapies, stem cells, engineered organ

## Abstract

Loss of organ and tissue due to injuries or diseases led to the development of regenerative therapies to decrease reliance on organ transplantations. It deals with employing the self-renewal ability of stem cells to differentiate into numerous lineages to assist in providing effective treatment for a range of various injuries and diseases. Regenerative engineering of organs or tissues represents an ever-expanding field that is aimed at developing biological replacements for dysfunctional organs or injured tissues. The critical issue, however, with the engineering of organs outside the human body is the insufficient availability of human cells, the absence of a suitable matrix with the same architecture and composition as the target tissue, and the maintenance of organ viability in the absence of the blood supply. The issue regarding the maintenance of the engineered organ viability can be solved using bioreactors consisting of mediums with defined chemical composition, i.e., nutrients, cofactors, and growth factors that can successively sustain the target cell’s viability. Engineered extracellular matrices and stem cells to regenerate organs outside the human body are also being used. Clinically, various adult stem cell therapies are readily under practice. This review will focus on the regeneration of organs through various types of stem cells and tissue engineering techniques.

## Introduction and background

Stem cells are undifferentiated cells having self-renewal properties found in the body that have the ability to differentiate into different types of cells with specialized functions [1]. Due to their pluripotency, differentiation, and self-regeneration property, they have become important for the treatment of a wide variety of diseases that include traumatic brain injury, stroke, Alzheimer’s disease, Parkinson’s disease, spinal cord injury, deafness, baldness, blindness, wound healing, myocardial infarction, rheumatoid arthritis, osteoarthritis, muscular dystrophy, and diabetes [2]. Research in stem cells offers not only unprecedented opportunities for the development of new ways and systems of tackling diseases but also paves the way for exploring fundamental questions and needs of humans.

Along with the traditional hematopoietic stem cell technique, organ transplantation has also become clinically important for kidney or liver failure [3]. However, the main obstacle in the organ transplantation program is the intense shortage of donor organs. In the present era, researchers have been finding ways for the successful establishment of the whole organ using stem cells, some of which include the generation of the organ using a single adult tissue stem cell, a blastocyst complementation system, decellularization and recellularization of bio-scaffold, a combinatorial technique employing stem cells and tissue engineering [2].

The recently introduced pluripotent stem cell technology is a successive advancement in the field of stem cell biology as it allows the generation of pluripotent stem cells in patients from their own blood cells or skin cells. Induced pluripotent stem cells can then undergo reprogramming to multiply and produce mature cells of high quality for cellular therapy. Moreover, no stimulation of unwanted immunogenic reactions and no immunosuppression would be needed. Through this technology, patients suffering from organ failure and waiting for transplants can be helped [2].

## Review

Generation of a functional organ from a single adult tissue stem cell

For the demonstration of the true stem cells in a given tissue, it is necessary for a single stem cell purified from tissue to generate the entire organ. The present clinical techniques successfully demonstrate the generation of prostate and mammary glands from a single adult tissue stem cell in vivo [4].

Generation of a Mammary Gland From a Single Adult Tissue Stem Cell

According to two independent reports, single stem cells isolated from the mammary glands of an adult mouse have the ability to produce secretory mammary glands when transplanted in the fat pad in mice. The capability of extensive growth during puberty and a third phase of expansion as well as contraction during pregnancy under the regulatory control of estrogen provided evidence that the mammary glands contain stem cells [5]. However, there was no isolating technique for mammary stem cells due to a lack of defined markers. This remained unproven until the work of some clinical specialists isolating mammary stem cells by the use of specific cell-surface markers (Lin-, CD29hi, CD24+) via FACSAria flow cytometry was successful [5]. Their research demonstrated that Lin-, CD29hi, and CD24+ mammary cells have the capability of in vitro formation of a sphere and repopulating all mammary epithelial cells after the transplantation into the fat pad. The insertion of a lineage tracer in the stem cells was an important step performed by the investigators so that they can follow their ultimate phenotypes in vivo [5]. The marking of a single cell of the Lin-CD29hiCD24+ population within the mouse mammary gland with a LacZ report transgene can result in the in vivo generation of a functional mammary gland. Notably, the cell that has been transplanted not only contributes to both myoepithelial as well as luminal lineages but also contributes to the generation of lobuloalveolar units that produces milk during pregnancy. This was an important discovery showcasing the multilineage differentiation capacity of a single adult tissue stem cell for the generation of a functional organ in vivo [5].

Generation of a Functional Prostate Gland From a Single Adult Tissue Stem Cell

By employing an assay for in vivo colony formation and transplantation approach of an in vivo renal capsule, it is possible to generate a functional prostate gland by isolating the single stem cell of an adult mouse prostate epithelium and utilizing its pluripotent ability. The existence of prostate stem cells was postulated because of their self-regenerating capacity following the deprivation of androgen and replacement. Due to cellular death after castration occurring in the luminal part of the cell, cell multiplication dominantly occurs in the basal compartment following the replacement of androgen. It is believed that the stem cells are present in the basal compartment of the cell and have the ability to repopulate the entire epithelial cells of the prostate [6]. For the generation of the prostate gland, first, for the prostate stem cells, the identification of a new marker CD117 is done. Identification is based on six features such as (i) the presence of a marker predominantly in the prostate’s proximal region; (ii) the marker should be expressed in the basal cell population; (iii) after castration, it should be upregulated and returned to its normal levels after androgen replacement; (iv) only CD117+ cells should have the ability to form in vivo colony structures with lumen; (v) second and third-generation CD117+ cells should have regeneration capability; and, lastly, (vi) the markers should have the ability to generate prostate epithelial glandular structures in vivo [6].

For the demonstration of the ability to generate a prostate structure by a single stem cell, cell sorting was done from the stem cell population that expressed multiple stem cell markers such as Sca-1+CD133+CD44+CD117+ into separated wells of a well plate having 96 wells. Subsequently, their verification was done under a microscope and then they were combined with embryonic urogenital sinus mesenchymal cells (rUGM) of a rat and transplanted into the renal capsule of athymic nu/nu mouse hosts. After three months when the kidneys were removed and the fate of the grafted cells was analyzed, out of 98 transplants of single cells, about one-seventh of Sca-1+CD133+CD44+CD117+ grafts demonstrated epithelial tubes with a branching pattern composed of luminal, prostatic, basal, and neuroendocrine lineages on immunocytochemical and histological examination [5]. The successful generation of prostate and mammary glands from the implantation of single stem cells in mice raised the possibility of further successful generation of mammary and prostate glands in humans that have lost these glands due to cancer [7].

According to some reports, some tissues contain cancer stem cells that have the ability to become tumors [7]. These cancer stem cells are believed to be derived from normal stem cells that have been altered due to some mutation or misplacement of tumor suppressor genes. Although the generation of the prostate gland was successful, this research may also aid in the marker identification for cancer stem cells that can be helpful in the efficient prostate cancer treatment in humans. This study not only provided a guideline for the reconstruction of mammary and prostate glands but also provided a foundation for the identification of cancer stem cells of mammary and prostate glands by the potential use of single stem cells clinically [7].

Generation of Heart by Human-Induced Cardiomyocytes Derived From Pluripotent Stem Cells

The pluripotent stem cells derived from cardiomyocyte technology have been applied for the disease modeling of cardiotoxicity testing, drug discovery, cardiomyopathies (inherited), regenerative therapies, channelopathies, and for the study of cardiac development and metabolic abnormalities [8]. Studies applied on different model organisms demonstrate that the transforming growth factor, nodal, activin, and bone morphogenetic protein signaling pathways play a significant role in the development of the cardiovascular system and can direct differentiated methodologies for the generation of human-induced pluripotent stem cell-derived cardiomyocytes. Several small molecules and growth factors improve the efficiency and reproducibility of the human-induced pluripotent stem cells-derived myocyte technology in both suspension cultures as well as adherent cultures. The purification methods for cardiomyocytes for the generation of a cardiac organ can be achieved by non-genetic methods that include mitochondria-specific dyes, deprivation of glucose, fluorescent probes, and cell surface markers [9].

Blastocyst Complementation System

Entire organs can be generated from pluripotent stem cells (PSCs) using blastocyst complementation in which PSCs are inserted into a blastocyst that is genetically modified for the development of a targeted organ. The PSCs get integrated into the developing embryo forming a chimeric animal in which the required organ generates from the inserted PSCs leading to the development of a targeted organ [10-12]. The need for organ transplantation therapies has resulted in the innovative idea of generating human organs in animal host bodies by injecting hPSC or human pluripotent cells into the blastocyst of animals. Studies have been conducted to test this technique through the creation of human-animal chimeras in embryos generated by humans and barnyard animals such as cattle and pigs [13]. This technique is further examined by integrating clustered regularly interspaced short palindromic repeats (CRISPR)-Cas9 gene modification with chimeric embryos for the generation of organs. Despite its potential, it should be noted that embryos have little tolerance for mistakes and speciation is a difficult endeavor. In addition, the formation of chimeras is experimentally low and requires resources and dedication.

Chimera Formation in Rodent Species Using Clustered Regularly Interspaced Short Palindromic Repeats With Interspecies Blastocyst Complementation

The utilization of chimeras for organ generation has been considered for some time and examples exist of chimeric embryos generated in rodent species [14-18]. Successful generation of chimeric embryos has only been achieved in mouse species, between mice and rats, and between sheep and goats by a combination of blastocyst cells from two species by injection or aggregation, into an embryo [19,20]. Experimentation has been done in rodent blastocysts using fluorescent markers for distinguishing host cells from donor cells, with pluripotent cells being labeled. These techniques follow the injection of rat stem cells labeled with marker into mouse blastocyst with subsequent transfer to the surrogate mouse. Even though chimerism is successful, quantification with polymerase chain reaction yields low results. However, this technique works as a baseline for the method of interspecies blastocyst complementation (IBC) that functions as a variation of the classical technique [21]. In this method, the blastocyst is modified with a mutation making it deficient for an organ and tissue, and this vacuum can then be filled with donor interspecific cells. For this purpose, the CRISPR-Cas9 gene editing tool is used [22]. This technique yields about 20% recorded mosaics which is considered an impressive outcome. As a result, this method has been used for generating rat pancreas in the mouse, rat heart, and eye by specific targeting of Pdx1, Nkx2.5, and Pax6. Despite being successful, the resulting organs are not “pure” even though they are enriched for rat cells. Moreover, even though Nks2.5 embryos obtained through CRISPR yield normal hearts, they have low survival rates. This indicates that IBC might not be compatible with life.

Human Chimerism in Embryos of Large Domestic Species

As techniques have been exploited for the generation of interspecific chimeras in rodents, chimerism was attempted between humans and domestic animals such as pigs and cows, larger animals being needed to “farm organs” as their size approaches that of humans. The aim of such methods has only been to determine possible cells that engraft during embryogenesis. hPSCs representing a range of pluripotent cells undergo testing for the ability to be able to survive in cattle or pig blastocysts. Naïve hPSCs represent the cells modeling the pre-implantation blastocyst cell mass, whereas primed hPSCs model the embryonic epiblast at the implantation stage. Insertion of primed cells results in no viable blastocysts, whereas when naïve hPSCs are inserted, they are detected 48 hours after incubation in the embryos maintaining pluripotent expression marker [23-25]. Modest proliferative ability is seen in experiments and this serves as the baseline for injecting human naïve cells into a pig embryo. A similar technique has been used to insert human naïve cells into pig blastocysts, with the chimerism lower than rat-mouse chimerism. To date, no viable animal-human chimeras exist due to blastocyst injection because of ethical boundaries and technical difficulties [26].

G*eneration of Exogenic Pancreas in Pigs Using Blastocyst Complementation System*

Regenerative medicine aims to generate functional organs from induced PSCs and functional pancreas, and kidneys have been generated in organ-disabled mice laying down the principle of generation of organs from PSCs in an organ-disabled embryo. The blastocyst complementation system can be utilized in pigs via the somatic cell cloning technique [11]. Organs generated using the complementation system have been derived from rodent species to produce human organs, and non-rodent animals are required that need to be made organ deficient and maintained at large numbers. Pigs are a potential candidate as somatic cell nuclear transfer (SCNT) is possible. For the development of the biliary system, Hes family bHLH transcription factor 1 (Hes1) expression is crucial, and its overexpression under the control of promoter pancreatic and duodenal homeobox 1 (Pdx1) results in pancreatic development inhibition [27]. Hence, apancreatic transgenic pigs can be generated. The intracytoplasmic sperm injection method is used for introducing the Pdx1-Hes1 construct in vitro into pig oocytes [28]. Transgenic pig fetuses were generated by transferring embryos, and some of the resulting embryos produce pancreatogenesis-disabled phenotype having a rudimentary pancreas. The fetus can then be used for the establishment of fibroblast cell cultures to be used as a donor cell for SCNT. The transgenic cells (Pdx1-Hes1) are used to produce clones via SCNT, and the cloned fetuses had the organ-disabled phenotype. To determine whether blastocyst complementation can be used to generate the pancreas in organ-disabled pigs, cloned transgenic embryos and cloned embryos with a gene encoding the Kusabira-Orange are used to generate chimeric embryos and then subsequently transferred to recipient gilts. It has been established that the pancreas generated from such gilts is normal in function and morphology, and it has been proven that PSC can occupy an organ-disabled niche resulting in a functional organ. The chimeric pigs can survive till adulthood providing a sperm source for large-scale production [11].

Blastocyst complementation for the generation of hematopoietic and vascular endothelial cells

For successful transplantation circumventing rejection, the generation of vascular endothelial cells within the organ is required. For the generation of these cells, a blastocyst complement system can be used using homozygous mutant blastocyst with vascular endothelial growth factor 2, the mutation that is lethal at embryonic days 8.5-9.5. The knockout chimeric mice homozygous Flk-1 can survive till the adult stage with injected PSC-derived endothelial and hematopoietic cells [29]. In mice, vascular endothelial cells start developing from the yolk sac at embryonic day 7.5 and are regulated by many factors such as vascular endothelial growth factor-2 (Flk-1). By its disruption, vasculogenesis can be inhibited [30,31]. During blastocyst complementation using Flk-1 mouse embryos, the hematopoietic stem cells (HSCs) develop from the complemented cellular niche (generated due to blastocyst complementation) that can then generate both PSC-derived hematopoietic and vascular endothelial cells [29].

Whole tissue generation using decellularization and recellularization techniques

Discarded human donor organs can be used for the provision of decellularized extracellular matrix (dECM) as suitable scaffolds for organ generation. Such dECM scaffolds can be used for organ engineering using hPSCs. Scaffolds have been created such as intact small intestine submucosa matrix generated by the mechanical removal of mesenteric tissues [32]. In similar ways, decellularization of other tissues such as heart valves, skin, vascular tissues, and bladder has been performed [32]. In addition, decellularization has been reported in many species such as pigs, rats, mouses, and humans [33]. This technique is based on the idea that extracellular matrix (ECM) proteins act as footprints from residing cells that can be distributed along the different organ sections [34]. The scaffold provides chemical and mechanical signals that aid cell differentiation, adhesion, and proliferation during recellularization [35].

Decellularization removes all the cellular content from the organ or tissue while simultaneously maintaining the ECM integrity and composition resulting in ECM scaffolds that retain biological and mechanical properties. The technique of perfusion decellularization delivers the agents throughout the organ while the immersion-based technique results in the agents of decellularization entering the organ through diffusion when the organ is submerged. The major difficulty in organ generation is the ability to position the organ inside its specific compartments, and for this cell seeding techniques into decellularized scaffolds require bioreactors that depend on the organ itself [36,37]. Seeding methods followed by vascular and non-vascular delivering routes have been used to introduce cells into the scaffolds. This method has been successful for the recellularization of liver, lung, heart, and kidney parenchyma [38].

To date, research regarding the recellularization of whole organs from decellularized scaffolds has been limited to small animal models because the problem with decellularizing is incomplete vasculogenesis and deficient parenchyma generation of the organ [39]. For humans, early works on organ generation focused on fetal cells from target organs that were seeded into decellularized scaffolds and maintained their phenotype. Recently, whole generation of human hearts from the recellularization of scaffolds has been conducted using human-induced pluripotent stem cell-derived cardiomyocytes (hiPSC-CMs). Human and mouse lung scaffolds have also been repopulated with cells from hiPSCs [40].

Synthesis of artificial blood vessels on microfluidic compartments

Blood vessels are the essential core of the organ system within the human body that act as a tubular channel connecting various tissues, cells, organs, glands, etc. The formation of artificial blood vessels was made possible by constructing a transferable culture of a vascular network module. But the question arises how can such an artificially produced network act as a natural and physiologically relevant unit within a microfluidic platform? Microfluidics is a field that deals with fluids on a micro level, which allows the in vitro model projection of a body on a single device under optimum conditions [41].

Lung fibroblasts (LFs) are the tissues present in lung airways which when combined with endothelial cells together can formulate in vitro network of blood vessels. These vessels possess the ability to communicate with other cells at short distances to induce some change and are capable of forming tubular structures. The focus for this setup was the paracrine effect inducing cell separation. The most suitable endothelial cells for this purpose are cells from the fetal umbilical vein that supplies the growing fetus with maternal blood. In this micro-scale production of vessels, the LFs were loaded on a media acting as a barrier providing a region purely of EC-forming capillaries. The continuous secretion of growth factor from LF facilitates growth through a hydrogel barrier which is an intrinsic property of tissue with high toughness and self-sealing ability. It has high tissue adhesiveness and cell affinity and integrates with surrounding tissues. After 10 days in a hydrogel, 95% of fibroblast seemed alive with the hydrogels providing a medium just like an ECM. The cell grows and divides rapidly, and the number of cell divisions can be controlled because it depends on the size and diameter of the loop [42].

Microfluidics generates in vitro vascular system with various improved characteristics similar to the natural vascular system by utilizing umbilical cells

Advancements in biological sciences have always contributed to existing research for better outputs. Correspondingly, blood vessels engineered by co-culturing LFs and endothelial cells showed a tight cell-to-cell junction, thus decreasing the permeability of the network. In this way, the single surface layer is completely covered by a group of cells to maintain rigidity and closeness, forming a packed monolayer. Thus, a texture with only a few local contact points for the exchange of biomolecules is formed just as bodily vessels. The production of vessels in a more natural manner is done in this microfluidic compartment utilizing umbilical endothelial cells rather than opting for the previous artificial tube procedures, which has various benefits. The blood vessels within the human body have the ability to vary their diameter. Umbilical endothelial cells impart this feature to synthesized blood vessels that lacked in the artificial tubes of the early methods. In this way, these vessels can be selected as functionally the most appropriate ones and can be used in future body-on-a-chip research [41,42].

Nevertheless, the generation of blood vessels is not just the basic task of such approaches. Detecting the actual functionality of blood vessels is equally important, which is to allow the flow of biomolecules, cells, and different constituents through its fluid compartment, a property known as perfusibility. The lumen, the central hollow opening of a vessel, is a vital part of tubular blood vessels. As deduced by the study, the lumen comprises growth factors in smaller amounts compared to that at the edges making them comparatively flat at the center but widened and having differentiating openings toward the ends [25]. Yet, the vessel network could not survive even a day in case the lumen was disassembled. This unit of the blood vessel is made functional by passing a fibroblast loop containing biomolecules [25]. These biomolecules then quickly perfuse through the vessels, creating a perfusable blood vessel network. Moreover, the other cells in close proximity could easily contaminate each other; however, overcoming this hurdle is also feasible by loop insertion that renders the interruption of endothelial cells with LFs. The paracrine factors inducing growth in nearby blood vessels are very crucial as they depend on the type of cell for the development of these vessels. In this way, future biologists can use different cell types within hydrogel loops to generate the desired blood vessel. Hence, the main goal of future research must be to devise a system in which newly formed blood vessels could survive even after the removal of fibroblast cells [25]. Based on microfluidics, humans succeeded to form such a platform that can be used to replace vessels in the real human body. The module functions similarly to the blood vessels and has clinical applications for illnesses in which a lack of blood vessel supply to certain organs causes problematic conditions. From this, it is possible to save lives and to understand the working of the body artificially.

Ear cartilage reconstruction by tissue engineering

Tissue engineering faces numerous challenges in reconstructing, repairing, and regenerating the external auricle of the ear and formulating a native structure similar in composition and function to that of the auricular cartilage. This has been made possible in vitro and in vivo by a combination of chondrocytes with either stem cells or any other optimal regenerating source. With the advancement in the field of regenerative medicine and tissue engineering, several biological approaches are adopted to generate analogs of ear cartilage as an alternative, such as carved costal cartilage or porous polyethylene implants [43].

Engineered chondrocyte cell sheets can rectify auricular defects and microtia

As many patients suffer from microtia, also known as acquired auricle defect, attempts have been made to produce autologous costal cartilage* sculptures and transplantation, alloplastic implants, and prosthetic ears. However, a majority of such attempts cause optimal aesthetic consequences resulting in health-related issues. To rectify such complications various synthetic polymers are utilized for implanting healthy cells at defective cartilage sites. This restores the mechanical stability of 3D-engineered tissue grafts made up of chondrocytes. A few attempts have been successful in this; however, others face problems mainly because artificial cartilage does not prove to be biocompatible due to a low cell viability rate. Moreover, there is an accumulation of degradative components during in vitro tissue generation leading to inflammatory reactions and necrosis. The feasibility of human chondrocyte sheet culture was investigated to increase the viability of cultured tissues [44-47]. The in vitro tissue culture of chondrocytes secretes gelatin-rich extracellular membranes once high confluency is reached [44]. The tissue tremelloid is formed by both a monolayer and bilayer of cell sheets, illustrating raised fluidity and gelling characteristics, as observed by an inverted microscope. On day 12 of tissue culture, the chondrocyte sheets shrink and form a gelatinous chondroid mass by folding up sheets into thickened stacks, as determined by histological analysis. This mass was then implanted in mice and developed into mature cartilage within eight weeks that were bound by fibrous tissues. Further studies can be conducted to analyze the effect of multilayer tissue development on constructing large-sized cartilages [44]. The tissue-engineered cartilage formed in this way is biodegradable, biocompatible, and a highly porous polymer mesh impregnated with isolated chondrocytes so that when this mesh is impregnated in the target body, the chondrocyte cells multiply and amplify into healthy matrix promoting the development of mature cartilages, which adopts the morphological characteristics of the biodegradable polymer mesh [47].

The critical role of periostin in shape retention of tissue-engineered cartilage after development

Periostin (PN) is a component abundant in fibrous tissues which plays a role in the maturation and retention of the shape of tissue-engineered cartilage by bringing about conformational changes in collagen molecules. PN bridges and stabilizes adjacent collagen fibrils during the process of fibril fusion. It also binds to other extracellular proteins by the N-terminal cysteine-rich domain combined with fibronectin and by Fasciclin 1 domain combined with tenascin C and bone morphogenetic protein 1. The effect of PN was studied by transplanting tissue-engineered cartilage in environmental conditions that lacked PN, producing irregularly shaped cartilage. Similarly, in an in vitro assay, the 3D culture embedded within the collagen gel that contained premixed PN enhanced chondrogenesis. This is because the PN-mediated collagen structure improves the mechanical strength of nearby fibrous tissue and activates chondrocyte extracellular signaling by interstitial fibrous tissues [46].

## Conclusions

Regenerative engineering is a rapidly growing field that harnesses advancements made in the field of tissue engineering with stem cell biology, materials science, and developmental biology. There has been successful production of clinically relevant therapies and technologies that improve patient outcomes and will continue to do so. Despite holding great promise, our abilities to employ stem cell therapies for organ regeneration are limited. To this end, preclinical research needs to focus on ways to elucidate the molecular mechanisms of differentiation and incorporation into target organs or tissues, factors affecting the proliferative and immunomodulatory effects, tissue distribution, characterization of cell markers, etc. Despite shortcomings associated with stem cell therapies or technologies, nanotechnology and biomedical engineering will be beneficial in overcoming them. Unfortunately, biomaterials can also be toxic once inside the human body, and hence, developing methods to increase the biocompatibility of materials is critical. Overall, new techniques should be developed for stem cell engineering such as the development of new materials, novel structures, and materials to better understand the interactions between the human body and the biomaterials used.
